# Influence of Early Enteral Nutrition on Clinical Outcomes in Neurocritical Care Patients With Intracerebral Hemorrhage

**DOI:** 10.3389/fneur.2021.665791

**Published:** 2021-04-20

**Authors:** Jianhua Peng, Bastian Volbers, Maximilian I. Sprügel, Philip Hoelter, Tobias Engelhorn, Yong Jiang, Joji B. Kuramatsu, Hagen B. Huttner, Arnd Dörfler, Stefan Schwab, Stefan T. Gerner

**Affiliations:** ^1^Department of Neurology, University Hospital Erlangen-Nuremberg, Erlangen, Germany; ^2^Department of Neurosurgery, The Affiliated Hospital of Southwest Medical University, Luzhou, China; ^3^Department of Neuroradiology, University Hospital Erlangen-Nuremberg, Erlangen, Germany

**Keywords:** intracerebral hemorrhage, nutrition, perihemorrhagic edema, prognosis, neurological intensive care

## Abstract

**Objective:** Early enteral nutrition (EEN) represents the current standard of care for patients treated in general intensive care units (ICU). Specific nutritional recommendations for patients receiving dedicated neurocritical care are not established. This study investigated associations of EEN with clinical outcomes for patients suffering from intracerebral hemorrhage treated at a neurological ICU (NICU).

**Methods:** This retrospective cohort study included patients admitted to the NICU with atraumatic ICH over a 4-year period. Nutritional data, demographic, clinical, radiological, and laboratory characteristics were assessed. EEN was defined as any enteral nutrition within 48 hours after admission. Comparisons were undertaken for patients with EEN vs. those without, further propensity score (PS) matching (caliper 0.2; one: many) was used to account for baseline imbalances. Primary outcome was the modified Rankin Scale (0–3 = favorable, 4–6 = unfavorable) at 12 months, secondary outcomes comprised perihemorrhagic edema (PHE) volume, infectious complications during the hospital stay, and mRS at 3 months, as well as mortality rates at 3 and 12 months.

**Results:** Of 166 ICH-patients treated at the NICU, 51 (30.7%) patients received EEN, and 115 (69.3%) patients received no EEN (nEEN). After propensity score matching, calories delivered from enteral nutrition (EEN 161.4 [106.4–192.3] kcal/day vs. nEEN 0.0 [0.0–0.0], *P* < 0.001) and the total calories (EEN 190.0 [126.0–357.0] kcal/day vs. nEEN 33.6 [0.0–190.0] kcal/day, *P* < 0.001) were significantly different during the first 48 h admitted in NICU. Functional outcome at 12 months (mRS 4–6, EEN 33/43 [76.7%] vs. nEEN, 49/64 [76. 6%]; *P* = 1.00) was similar in the two groups. There were neither differences in mRS at 3 months, nor in mortality rates at 3 and 12 months between the two groups. EEN did not affect incidence of infective complications or gastrointestinal adverse events during the hospital stay; however, EEN was associated with significantly less extent of PHE evolution [maximum absolute PHE (OR 0.822, 95% CI 0.706–0.957, *P* = 0.012); maximum relative PHE (OR 0.784, 95% CI 0.646–0.952, *P* = 0.014)].

**Conclusion:** In our study, EEN was associated with reduced PHE in ICH-patients treated at a NICU. However, this observation did not translate into improved survival or functional outcome at 3 and 12 months.

## Introduction

Intracerebral hemorrhage (ICH) accounts for up to 10–15% of all stroke cases with mortality rates up to 61% at 1 month (1, 2). Given severe neurological impairments and impaired consciousness, ICH survivors often require admission to the intensive care unit (ICU) or neurological ICU (NICU) (3). Current guidelines for critical patients recommend that practitioners consider initiating enteral feeding after admission to the ICU (within 48 h) (4). However, detailed management or any associated effects of nutritional support for stroke patients in the NICU have not been established yet.

Sufficient and early intake of energy by enteral feeding is thought to help attenuate the metabolic response to stress, favorably modulate immune responses, and diminish complications (5). So far, the effects of enteral nutrition on perihemorrhagic edema (PHE) dynamics, mortality, and functional outcome in neurocritical care patients with ICH have not been established. The present study investigated the association of early enteral nutrition (EEN) with (1) functional outcomes and (2) mortality at 12 months, (3) PHE evolution, as well as (4) infectious complications and gastrointestinal adverse events during the hospital stay in ICH patients admitted to NICU.

## Materials and Methods

### Study Design and Patient Selection

This retrospective cohort study included patients with spontaneous ICH who were admitted to the NICU between January 2012 and December 2015 from our prospective single-center UKER-ICH registry (Universitaetsklinikum Erlangen Cohort of Patients with Spontaneous Intracerebral Hemorrhage; clinical-trials.gov NCT03183167). Detailed information and methods of UKER-ICH have been published previously (6). The collection of data for the UKER-ICH registry was approved by the ethics committee of the local university (IRB No. 115_17B). Consent for follow-up assessment was obtained by either the patient, his/her legal representative or the closest relative.

We excluded patients with secondary causes of ICH, such as tumor, trauma, arteriovenous malformation, aneurysm, or acute thrombolysis (6). Furthermore, patients staying NICU <72 h, as well as patients receiving early care limitations, were excluded from the final analysis.

### Assessment of Clinical and Imaging Parameters

As described previously, we assessed demographic data, medical history, laboratory data, infectious complications, and clinical parameters through review of institutional databases and patient's medical charts (6). Imaging parameters were assessed by evaluating all available imaging scans during the hospital stay as described previously (6). To account for various time points of control CTs, we categorized follow-up imaging according to time frames (days 1, 2–3, 4–6, 7–9, 10–12 and 13–15) with day 1 defined as the day of admission. PHE was assessed using an established and validated semi-automatic threshold-based algorithm by calculation of the maximum absolute PHE, which was defined as peak PHE volume during hospital stay, and the maximum relative PHE (rPHE) defined as the ratio of peak PHE volume divided by the final ICH volume (7, 8).

### Nutrition Data Assessment

We assessed the nutrition data from the integrated care manager system (ICM; Drägerwerk AG Co. KGaA, Lübeck). The nutrition data were assessed for up to 20 days or until the patient discontinued enteral nutrition, died, or was discharged from the NICU, whichever occurred first. We defined EEN as any enteral tube feeding within 48 h after admission to our NICU. Otherwise, patients were classified into the no early enteral nutrition (nEEN)-group if no enteral feeding was performed within the first 48 h. Target calorie goals were determined by indirect calorimetry or weight-based predictive equation (age <30 years: 25 kcal/kg ideal bodyweight; age from 30–70 years: 22.5 kcal/kg ideal bodyweight; age>70 years: 20 kcal/kg ideal bodyweight; each of that multiplied with a factor of 1.2 for NICU-patients). During the NICU-stay, daily records were assessed regarding all procedures of enteral nutrition, parenteral nutrition, fluid balance, vomiting, and defecation. In addition to the calories from enteral nutrition or parenteral nutrition, other energy sources such as oral intake were evaluated by nursing records. Constipation was defined as failure to pass stool within 72 h of admission to the NICU or failure to pass stool for three consecutive days during the NICU stay (9). Diarrhea was defined as three or more loose, or liquid stools per day for two consecutive days (10).

### Primary and Secondary Outcomes

Primary outcome was defined as the proportion of patients with unfavorable outcome at 12 months [modified Rankin Scale (mRS) score = 4–6 (11)] comparing EEN and nEEN-patients. Follow-up data were evaluated 12 months after onset of ICH by mailed questionnaires, telephone interviews, or chart review using the mRS score (performed by trained and certified physicians; scores 0–6, higher scores indicating worse outcome and 6 indicating death). Secondary outcomes consisted of (1) mRS at 3 months, (2) mortality rates at 3 and 12 months, as well as (3) PHE volume evolution, and (4) infectious complications and gastrointestinal complications (constipation or diarrhea) during hospital stay.

### Statistical Analyses

Statistical analyses were performed by SPSS version 21.0 (IBM; ibm.com/analytics) and R 3.3.1 (r-project.org) as described previously (12). According to data-distribution assessed using the Kolmogorov-Smirnov test, we compared normally distributed data (expressed as mean ± SD) using the Student *t* test; otherwise, we used the Mann Whitney *U* test presented as median (interquartile range) for non-normally distributed data. Frequency distributions of categorical variables [presented as counts (percentage)] were compared by Pearson χ^2^ and Fisher exact tests. Statistical significance was set at α = 0.05 both sided.

To account for baseline imbalances in relevant clinical parameters showing a statistical trend (*P* < 0.1) in prior univariate analysis between EEN and nEEN cohorts, we performed a propensity score (PS) matching (balanced, parallel nearest-neighbor approach, ratio 1:many, caliper 0.2) (11). The following variables were selected to generate the propensity score: Diabetes mellitus, Graeb score, mechanical ventilation, and the Glasgow Coma Scale (GCS) score on admission.

To determine associations between EEN and maximum absolute and relative PHE (both median-split) binary regression analyses (log-nominal with link-identity) were calculated. Associations were expressed as odds ratios (ORs) with the corresponding 95% confidence interval. Adjustment for ICH-volume was undertaken for absolute PHE considering the reported impact of ICH-surface on absolute PHE development (6).

## Results

**Figure 1:**
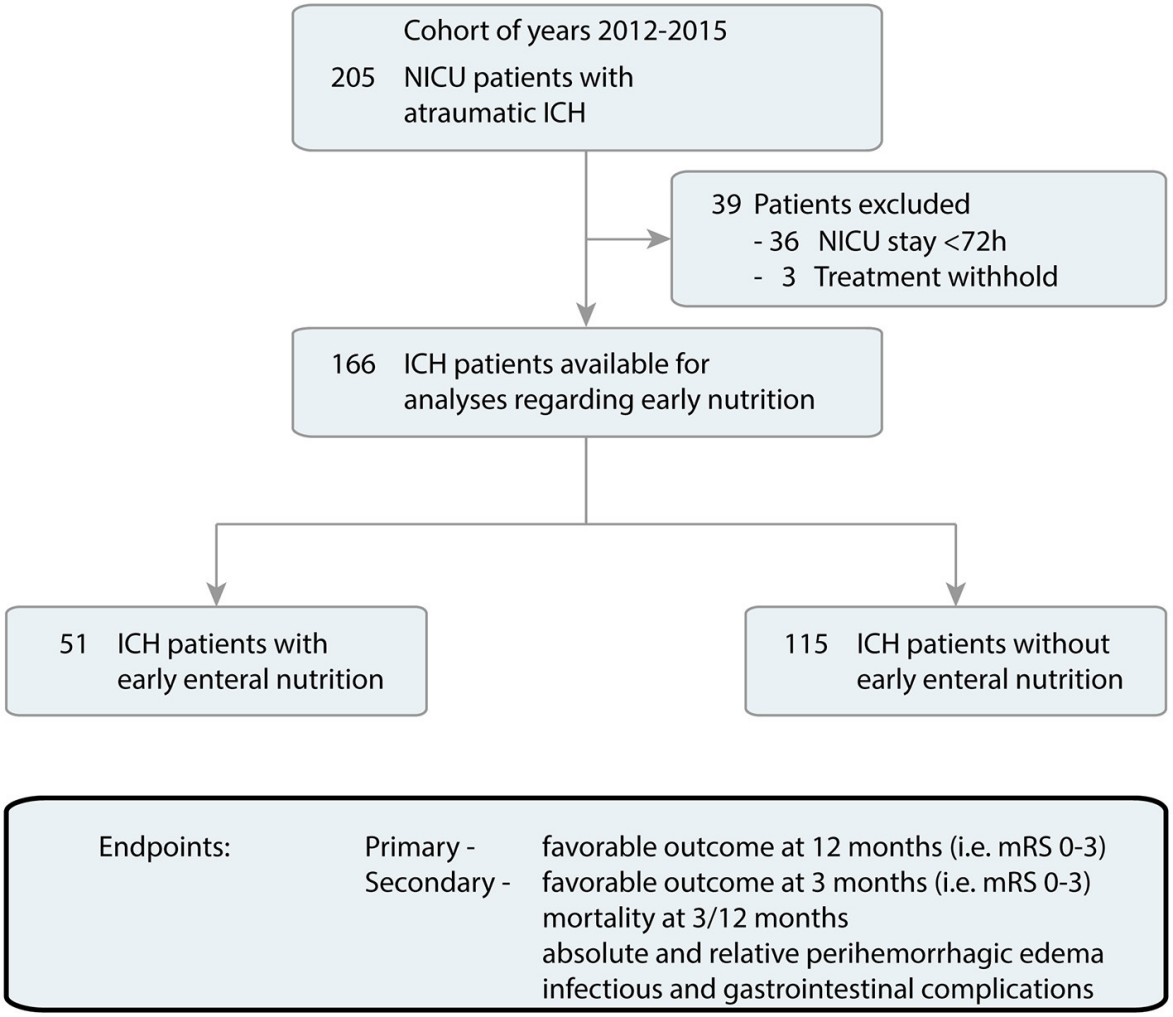
Flow chart of included patients. Overall, The UKER-ICH provided data of 205 patients with ICH admitted in NICU (years 2012–2015). After exclusion of 39 patients because of NICU stay <72 h or/and treatment withhold, 51 patients with EEN and 115 patients with nEEN remained for further data analyses. EEN, early enteral nutrition; ICH, intracerebral hemorrhage; mRS, modified Rankin Scale; nEEN, no early enteral nutrition; NICU, neuro-intensive care unit.

Over a 4-year period, 205 NICU patients with primary ICH were treated at our NICU and screened for eligibility. After exclusion of 39 patients because of NICU stay <72 h or/and treatment withhold, 51/166 (30.7%) EEN, and 115/166 (69.3%) nEEN treated ICH patients remained for final analyses (Figure 1).

### Clinical and Radiologic Characteristics

Baseline, hematoma, and treatment characteristics are provided in Table 1 comparing EEN vs. nEEN-patients. Compared to nEEN, patients with EEN had more frequently history of diabetes mellitus [EEN 23/51 (45.1%) vs. nEEN 29/115 (25.2%), *P* = 0.018] and a worse clinical status on admission [GCS, median (IQR): EEN 7 (3–12) vs. nEEN 12 (6–14), *P* < 0.001; National Institute of Health Stroke Scale (NIHSS), EEN 22 (13–38) vs. nEEN 14 (7–29), *P*=0.002; ICH Score, EEN 2 (2–3) vs. nEEN 2 (1, 2), *P* = 0.005]. There was no significant difference regarding ICH-volume and location among both groups. EEN-patients had intraventricular hemorrhage (IVH) at a higher rate and extent compared to patients without EEN [IVH, No. (%): EEN 39/51 (76.5%) vs. nEEN 61/115 (53%), *P* = 0.006; Graeb Score, median (IQR): EEN 4 (1–8) vs. nEEN 1 (0–5), *P*=0.002]. EEN patients required more often mechanical ventilation [EEN 47/51 (92.2%) vs. nEEN 71/115 (63.4%), *P* < 0.001] and placement of external ventricular drain [EEN 39/51 (76.5%) vs. nEEN 50/115 (43.5%), *P* < 0.001; Table 1].

**Table 1:** Characteristics of ICH patients with EEN vs. nEEN.

**Patients with ICH (*n* = 166)**	**EEN (*n* = 51)**	**nEEN (*n* = 115)**	***P*-value**
Age, median (IQR), y	72 (61–77)	73 (59–80)	0.494
Female sex, No. (%)	22 (43.1%)	45 (39.1%)	0.732
**Prior comorbidities, No. (%)**			
Premorbid mRS, median (IQR)	1 (0–3)	0 (0–1)	0.191
Hepatic dysfunction	3 (5.9%)	12 (10.4%)	0.558
Renal failure	11 (21.6%)	22 (19.1%)	0.833
Arterial hypertension	47 (92.2%)	108 (93.9%)	0.738
Diabetes mellitus^†^	23 (45.1%)	29 (25.2%)	**0.018**
Prior ischemic stroke	11 (21.6%)	18 (15.7%)	0.380
Prior hemorrhagic stroke	7 (13.7%)	13 (11.3%)	0.796
Antiplatelet medication	14 (27.5%)	34 (29.6%)	0.854
Oral anticoagulation	12 (23.5%)	25 (21.7%)	0.841
**Admission status, median (IQR)**			
Glasgow coma scale^†^	7 (3–12)	12 (6–14)	**<0.001**
NIHSS^†^	22 (13–38)	14 (7–29)	**0.002**
ICH Score^†^	2 (2–3)	2 (1–2)	**0.005**
CHADS VASc score	3 (2–5)	3 (2–5)	0.532
HAS bled score	2 (2–4)	2 (2–4)	0.986
**Imaging, median (IQR)**			
Initial ICH volume, ml	18.3 (6.9–37.3)	12.9 (3.93–29.6)	0.110
**ICH location, No. (%)**			
Deep	31 (60.8%)	61 (53%)	0.400
Lobar	11 (21.6%)	36 (31.3%)	0.263
Cerebellar	7 (13.7%)	13 (11.3%)	0.796
Brainstem	2 (3.9%)	5 (4.3%)	0.632
Intraventricular hemorrhage, No. (%)^†^	39 (76.5%)	61 (53%)	**0.006**
Graeb Score^†^	4 (1–8)	1 (0–5)	**0.002**
**Clinical parameter**			
Mechanical ventilation, No. (%)^†^	47 (92.2%)	71 (63.4%)	**<0.001**
EVD, No. (%)^†^	39 (76.5%)	50 (43.5%)	**<0.001**

*EEN, early enteral nutrition; EVD, external ventricular drain; HAS-BLED, Hypertension, Abnormal Renal and Liver Function, Stroke, Bleeding, Labile INR-Measures, Elderly (age >65 y) and Drugs or Alcohol; ICH, intracerebral hemorrhage; IQR, interquartile range; mRS, modified Rankin Scale; nEEN, no early enteral nutrition; NIHSS, National Institutes of Health Stroke Scale*.

† *Significant differences are highlighted in bold*.

### Nutritional Characteristics

To specifically compare outcome among ICH patients with EEN vs. nEEN, we adjusted for the previously mentioned baseline confounders using a PS matching. After PS matching (Supplementary Table 1), 47 ICH patients with EEN and 67 ICH patients with nEEN were available for further analyses without significant differences in relevant parameters.

Nutritional characteristics of EEN- and nEEN-patients are provided in Table 2. Patients received the enteral nutrition for a median of 12 days (4–15) in the EEN group and 9 days (2–13) in the nEEN group, respectively (*P* = 0.033). Supplementary parenteral nutrition was initiated earlier [nEEN (3(2–4) vs. EEN 4(3–7), *P* = 0.033] and lasted longer [nEEN (3(0–8) vs. EEN 0(0–3), *P* = 0.005] in the nEEN group compared with the EEN group. During the first 48 h at NICU, calories delivered from enteral nutrition [EEN 161.4 (106.4–192.3) kcal/day vs. nEEN 0.0 (0.0–0.0), *P* < 0.001] and calories delivered from enteral nutrition plus other sources [EEN 190.0 (126.0–357.0) kcal/day vs. nEEN 33.6 (0.0–190.0) kcal/day, *P* < 0.001] were significantly different between both groups in favor of EEN (Table 2). During hospital course, there was no significant difference in nutrition-related laboratory parameters among both groups.

**Table 2:** Nutrition characteristics of PS-matched patients.

**PS-matched patients with ICH (*n* = 114)**	**EEN (*n* = 47)**	**nEEN (*n* = 67)**	***P*-value**
**Start of nutrition, day since admission, median (IQR)**
Enteral nutrition^†^	2 (2–2)	3 (3–4)	**<0.001**
Parenteral nutrition^†^	4 (3–7)	3 (2–4)	**0.033**
**Length of nutrition, days, median (IQR)**			
Enteral nutrition^†^	12 (4–15)	9 (2–13)	**0.033**
Parenteral nutrition^†^	0 (0–3)	3 (0–8)	**0.005**
Calorie target, kcal/day median (IQR)	1854.0 (1800.0–1854.0)	1854.0 (1854.0–1854.0)	0.757
**Nutrition within 1st 48h (IQR), kcal/day**			
Enteral calories^†^	161.4 (106.4–192.3)	0.0 (0.0–0.0)	**<0.001**
Parenteral calories^†^	0.0 (0.0–0.0)	33.6 (0.0–190.0)	**0.014**
Total calories^†^	190.0 (126.0–357.0)	33.6 (0.0–190.0)	**<0.001**
**Total Nutrition (IQR), kcal/day**			
Enteral calories	1102.0 (611.2–1463.7)	980.2 (598.0–1239.8)	0.071
Parenteral calories	0.0 (0.0–682.3)	447.3 (0.0–764.0)	0.172
Total calories	1316.4 (872.9–1550.6)	1386.7 (898.6–1539.3)	0.879
**Laboratory values at 48 h (Mean** **±** **SD)**			
Chloride, mmol/L	107.4 ± 5.2	106.7 ± 4.2	0.450
Potassium, mmol/L	4.2 ± 0.3	4.1 ± 0.4	0.109
Sodium, mmol/L	140.9 ± 4.6	139.7 ± 3.7	0.129
Urea nitrogen, mg/dl	37.0 ± 16.4	36.6 ± 16.4	0.896
Total protein, g/L	53.4 ± 5.5	55.2 ± 6.2	0.167
Osmolar, mOsmol/kg	300.3 ± 12.9	297.1 ± 11.1	0.200
**Laboratory values during NICU stay (Mean** **±** **SD)**			
Chloride, mmol/L	108.8 ± 5.2	107.9 ± 4.8	0.325
Potassium, mmol/L	4.3 ± 0.3	4.2 ± 0.2	0.388
Sodium, mmol/L	143.0 ± 4.3	141.9 ± 4.5	0.204
Urea nitrogen, mg/dl	52.4 ± 26.2	46.2 ± 20.3	0.162
Hemoglobin, g/dl	13.5 ± 1.9	13.4 ± 2.5	0.772
Total protein, g/L	52.7 ± 5.7	53.4 ± 5.0	0.460
Osmolar, mOsmol/kg	306.6 ± 13.3	303.7 ± 13.2	0.260
Fluid balance (IQR), ml/day	+340.2 (214.3–732.6)	+526.7 (174.5–769.1)	0.502
Median highest daily blood glucose concentration (IQR), mg/dl	180.1 (157.1–216.6)	176.3 (144.8–197.2)	0.140

*EEN, early enteral nutrition; ICH, intracerebral hemorrhage; IQR, interquartile range; nEEN, no early enteral nutrition; PS, propensity score*.

† *Significant differences are highlighted in bold*.

### Primary Outcome

**Figure 2:**
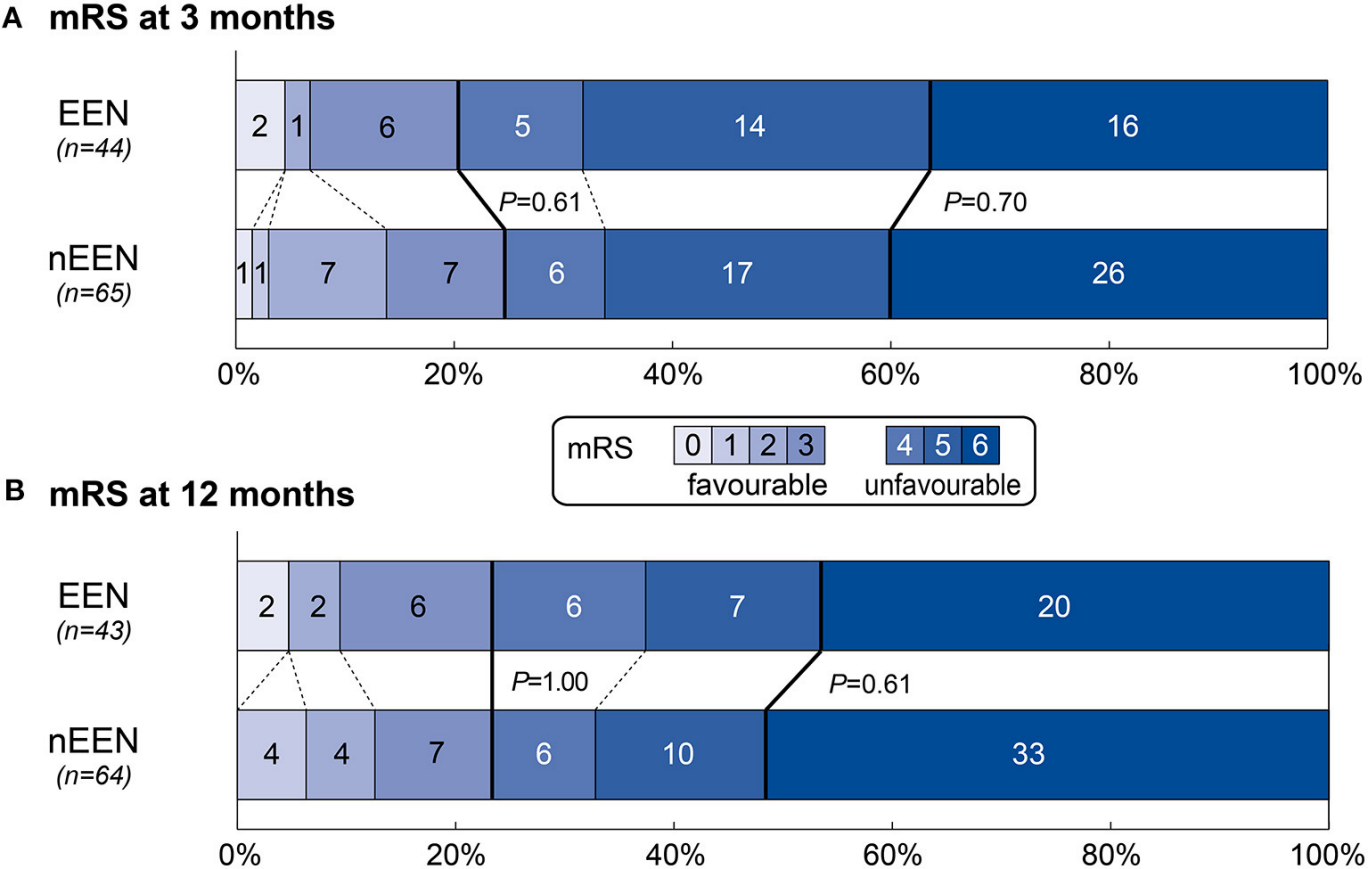
Distribution of mRS at 3 and 12 months comparing patients with EEN and nEEN. Presented are mRS-scores after 3 **(A)** and 12 **(B)** months comparing patients with EEN and nEEN after PS matching (matching parameter: diabetes mellitus, GRAEB score, mechanical ventilation and the initial Glasgow Coma Scale). The bold line separates favorable (mRS, 0–3) and unfavorable outcome (mRS, 4–6). *P*-values are provided for the proportion of patients with favorable outcome and in hospital death. EEN, early enteral nutrition; ICH, intracerebral hemorrhage; mRS, modified Rankin Scale; nEEN, no early enteral nutrition.

The distribution of mRS at 12 months is illustrated in Figure 2B. There was no difference regarding the proportion of patients achieving favorable outcome at 12 months [mRS, 0–3: EEN, 10/43 (23.3%) vs. nEEN, 15/64 (23.4%); *P* = 1.00; Table 3] between patients with EEN and nEEN.

**Table 3:** Outcomes and complications of PS-matched patients comparing EEN vs. nEEN.

**PS-matched patients (n=114)**	**EEN (n = 47)**	**nEEN (n = 67)**	***P*-value**
Length of hospital stay (IQR)	17 (10–22)	16 (12–25)	0.498
Length of NICU stay (IQR)	15 (8–20)	14 (8–22)	0.762
**Functional outcomes, No. (%)**			
mRS 0–3 at 3 months	9 (20.5%)	16 (23.4%)	0.610
Mortality at 3 months	16 (36.4%)	26 (40.0%)	0.699
mRS 0–3 at 12 months	10 (23.3%)	15 (23.4%)	1.000
Mortality at 12 months	20 (46.5%)	33 (51.6%)	0.610
**Perihemorrhagic edema, median (IQR)**			
Maximum absolute PHE during hospital stay, ml^†^	26.7 (6.5–39.5)	34.8 (8.5–58.4)	**0.021**
**Gastrointestinal outcomes**			
Regurgitation or vomiting, No. (%)	30 (63.8%)	39 (58.2%)	0.566
Median bowel movements per day (IQR)	0.9 (0.5–1.4)	0.9 (0.5–1.3)	0.895
Constipation, No. (%)	25 (53.2%)	29 (43.3%)	0.343
Diarrhea, No. (%)	28 (59.6%)	36 (53.7%)	0.570
Infectious complications, No. (%)	37 (78.7%)	52 (77.6%)	1.000
Pneumonia	23 (48.9%)	41 (61.2%)	0.250
Ventriculitis	4 (8.5%)	8 (11.9%)	0.758
Sepsis	14 (29.8%)	20 (29.9%)	1.000
Urinary tract infection	6 (12.8%)	7 (10.4%)	0.769
In-hospital mortality, No. (%)	12 (25.5%)	18 (26.9%)	1.000

*EEN, early enteral nutrition; EVD, external ventricular drainage; IQR, interquartile range; nEEN, no early enteral nutrition; NICU, neurological intensive care unit*.

† *Significant differences are highlighted in bold*.

### Secondary Outcomes

There was no significant difference regarding functional outcomes at 3 months [mRS, 0–3: EEN, 9/44 (20.5%) vs. nEEN, 16/65 (23.4%), *P* = 0.61; Table 3 and Figure 2A], and no significant differences in rates of mortality at 3 and 12 months [mortality at 3 months: EEN 16/44 (36.4%) vs. nEEN 26/65(40%), *P* = 0.70; mortality at 12 months: EEN 20/43 (46.5%) vs. nEEN 33/64(51.6%), *P* = 0.61; Table 3 and Figures 2A,B].

**Figure 3:**
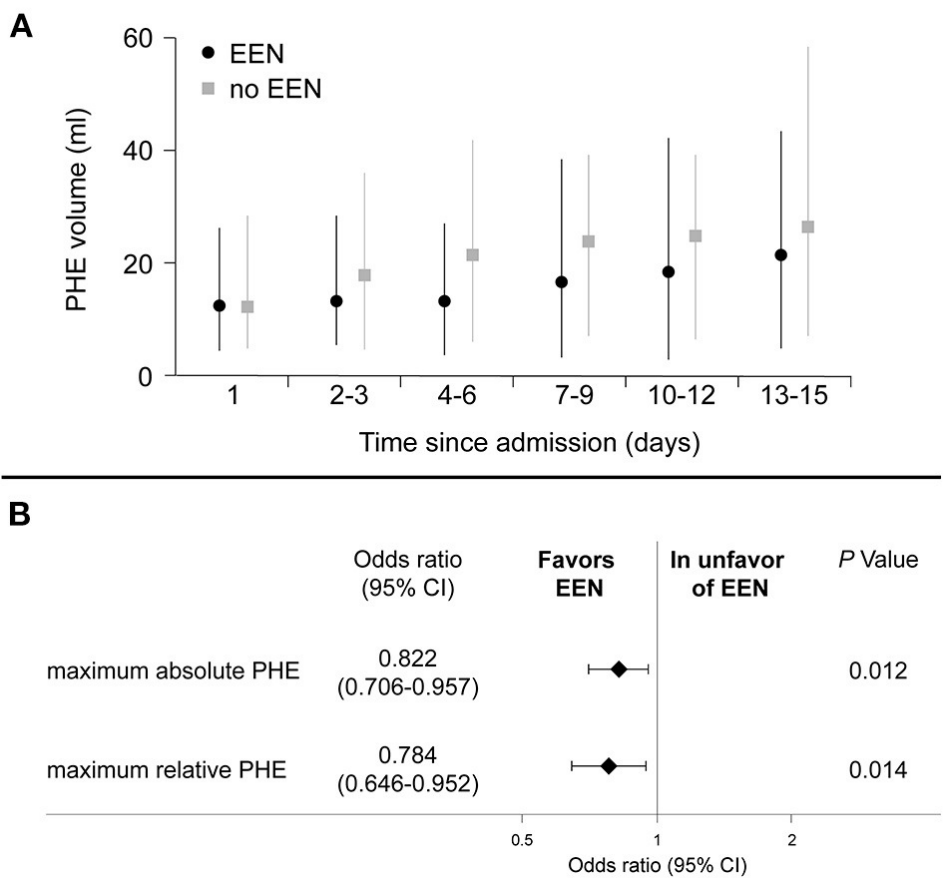
**(A)** Time course of perihemorrhagic edema and **(B)** associations with EEN. **(A)** Illustrated are the PHE volumes for each time-interval for ICH-patients with and without EEN for the PS-matched cohort. **(B)** Associations between EEN and maximum absolute and relative perihemorrhagic edema are provided. Binary logistic regression analysis (nominal with link-identity) was used. Associations are expressed as odds ratios and corresponding 95% confidence interval. Adjustment for ICH-volume was undertaken for absolute PHE considering the reported impact of ICH-surface on absolute PHE development. CI, confidence interval; EEN, early enteral nutrition; ICH, intracerebral hemorrhage; nEEN, no early enteral nutrition; PHE, perihemorrhagic edema.

The time course of absolute PHE is illustrated in Figure 3A. Highest median absolute PHE was observed between day 13 and 15 after admission in both groups. There were no significant differences regarding absolute PHE among EEN and nEEN patients at each time-point of imaging. However, maximum absolute PHE during hospital stay was significantly less in EEN- compared to nEEN-patients [median absolute PHE (IQR): EEN 26.7 (6.5–39.5) ml vs. nEEN 34.8 (8.5–58.4)ml; *P* = 0.021; Table 3]. In addition, regression analyses revealed significant associations between EEN and maximum absolute as well as relative PHE. In essence, EEN was associated with attenuated maximum absolute PHE (OR 0.822, 95% CI 0.706–0.957, *P* = 0.012) and maximum relative PHE (OR 0.784, 95% CI 0.646–0.952, *P* = 0.014) as shown in Figure 3B.

Regarding the safety outcomes, there were no differences in rates of constipation [EEN 25/47 (53.2%) vs. nEEN 29/67 (43.3%), *P* = 0.343] and diarrhea [EEN 28/47 (59.6%) vs. nEEN 36/67 (53.7%), *P* = 0.570] between both groups (Table 3). Further, we observed similar rates of infectious complications during the hospital stay among patients with and without EEN [EEN: 37/47 (78.7%) vs. nEEN 52/67 (77.6%); *P* = 1.000; Table 3].

## Discussion

The present study evaluated the associations of EEN with functional outcome, mortality, PHE evolution, as well as infectious complications in ICH patients treated at NICU. As key findings, we demonstrated that EEN was not associated with functional outcome or mortality at 3 or 12 months, and there were no differences regarding infectious or gastrointestinal complications during hospital stay. Yet, EEN was associated with a lower extent of PHE evolution during hospital stay in ICH-patients. Some aspects deserve attention.

Nutritional supplementation represents one of the cornerstones of supportive care in NICU. Enteral feeding is recommended to be started within 48 h for surgical and medical critical care patients (4) and is recommended for critically ill ICH patients (13). However, these recommendations have not been supported by studies specifically analyzing neurocritical care stroke patients, and were rather based on pathophysiological considerations extrapolated from general ICU patients. Our study now adds on to this discussion, as we systematically analyzed nutrition regimens specifically for neurocritical care ICH patients. Although our study was not powered to detect differences, we here did not find EEN, compared to no EEN, to significantly alter clinical outcomes, notably the rate of mortality or the proportion of patients with good functional outcome at follow-up.

However, our study did reveal reduced PHE evolution in patients who received EEN. There are several potential causal mechanisms underlying our findings. As reported elsewhere, secondary injury cascades and detrimental processes, including degradation of heme-products and neuro-inflammation post-ICH, are considered to contribute to PHE formation (8). Route and amount of nutrition is thought to help attenuate the metabolic response to stress and favorably modulate immune responses (4, 14). First, in our cohort, relative PHE in patients with EEN was lower than in patients without EEN, which may be partly explained by the reported anti-inflammatory mechanism of early enteral feeding. Second, malnutrition may lead to altered Na^+^-K^+^ ATPase activity, ATP depletion, and a rise in intracellular osmotic pressure, which result in blood–brain barrier (BBB) disruption and angioedema (15). Thus, at the acute phase of ICH-treatment, sufficient provision of energy seems essential to potentially attenuate BBB disruption and angioedema by maintaining mitochondrial metabolism.

However, despite its beneficial association with reduced PHE, EEN did not translate into improved clinical outcomes. This finding is in line with previous studies in which several treatment approaches targeting PHE-development failed to influence outcome reflecting the complex pathophysiology of PHE in patients with ICH (16–20). Further studies including extensive assessments of nutrition status are warranted to determine the detailed mechanisms of early enteral feeding on cerebral edema.

In line with available guidelines, we did not detect any safety issues of EEN in neurocritical care ICH patients. In our cohort, EEN did not increase the rates of infectious complications or gastrointestinal complications during the hospital stay. Instead, undernutrition may be associated with prolonged length of stay and mechanical ventilation, infection, and mortality (21). However, we could not find decreased rates of pneumonia or any other infections in patients treated with EEN, which might occur due to the small number of patients, and the unreached calorie targets in some patients. It is important to note that that caution should be taken to avoid early overfeeding and the resultant increase in risk of complications (22, 23). The optimal energy and protein target in the early phase of acute critical illness is currently unknown. In this retrospective cohort, we did not find a favorable effect of EEN on long-term functional outcomes of patients with ICH. But, due to its safety profile, potential protective effects on edema evolution and positive signals from trials investigating general ICU-patients the use of EEN seems reasonable in ICH patients. Further prospective and randomized studies are required to fully understand the effects of EEN especially in ICH patients requiring NICU-treatment.

Our study has several limitations mainly given its retrospective analysis and monocentric design. As a consequence, there was no *a-priori* defined assessments of nutrition status, complications, and follow-up brain imaging at certain time-points leaving some room for bias of our results. Despite the sophisticated statistical efforts to account for imbalances, generalizability of our findings may be limited due to selection bias. Further, the small sample size did not allow further subgroup-analyses or adjustments, for example, to adjust for treatment strategies to target PHE, such as the use of therapeutic hypotherma or osmotic agents. Due to its retrospective design, calories from other sources, i.a. medications, could not be completely counted.

## Conclusions

In our study, EEN was associated with reduced PHE in ICH-patients treated at a NICU. However, this observation did not translate into improved survival or functional outcome at 3 and 12 months.

## Data Availability Statement

The original contributions presented in the study are included in the article/Supplementary Material, further inquiries can be directed to the corresponding author/s.

## Ethics Statement

The collection of data for the UKER-ICH registry was approved by the ethics committee of the local university (IRB No. 115_17B). The patients/participants provided their written informed consent to participate in this study.

## Author Contributions

SG, JP, BV, and JK contributed to data analysis. SG, JP, BV, MS, JK, PH, TE, and AD contributed to data collection. SG, JP, MS, HH, AD, and SS contributed to study design. SG, JP, BV, and HH contributed to drafting initial manuscript. All authors contributed to manuscript revision.

## Conflict of Interest

The authors declare that the research was conducted in the absence of any commercial or financial relationships that could be construed as a potential conflict of interest.
