# Supplementary material for: Influence of Early Enteral Nutrition on Clinical Outcomes in Neurocritical Care Patients With Intracerebral Hemorrhage
Source: Front Neurol. 2021 Apr 20;12:665791. doi: 10.3389/fneur.2021.665791 (PMC8093818; doi:10.3389/fneur.2021.665791)
Supplement: Supplementary file 1 [file Table_1.docx]

**Influence of Early Enteral Nutrition on Clinical Outcomes in Neurocritical Care Patients with Intracerebral Hemorrhage**

Running title: Early enteral nutrition in ICH patients

**Jianhua Peng**, MD^1,2,*^ pengjianhua@swmu.edu.cn; **Bastian Volbers**, MD^1,*^ Bastian.Volbers@uk-erlangen.de; **Maximilian I. Sprügel**, MD^1^, Maximilian.Spruegel@uk-erlangen.de; **Philip Hoelter**, MD^3^, Philip.Hoelter@uk-erlangen.de; **Tobias Engelhorn**, MD^3^, Tobias.Engelhorn@uk-erlangen.de; **Yong Jiang**, MD^2^, jiangyong@swmu.edu.cn; **Joji B. Kuramatsu**, MD^1^, Joji.Kuramatsu@uk-erlangen.de; **Hagen B. Huttner**, MD/PhD^1^, Hagen.Huttner@uk-erlangen.de; **Arnd Dörfler**, MD^3^, Arnd.Doerfler@uk-erlangen.de; **Stefan Schwab**, MD^1^, Stefan.Schwab@uk-erlangen.de; and **Stefan T. Gerner**, MD^1^, Stefan.Gerner@uk-erlangen.de.

^1^ Department of Neurology, University Hospital Erlangen-Nuremberg, Germany.

^2^ Department of Neurosurgery, the Affiliated Hospital of Southwest Medical University, China.

^3^ Department of Neuroradiology, University Hospital Erlangen-Nuremberg, Germany.

^*^ These authors contributed equally to this manuscript

**Corresponding Author:**

Stefan T. Gerner, Dpt. of Neurology, University Hospital Erlangen-Nuremberg;

Email: Stefan.Gerner@uk-erlangen.de

**Supplemental digital Table 1:** Characteristics of ICH patients with EEN versus nEEN after PSM

| **PS-matched patients with ICH (n=114)** | **EEN (n=47)** | **nEEN (n=67)** | ***P* Value** |
| --- | --- | --- | --- |
| Age, median (IQR), y | 72 (60-76) | 74 (65-80) | 0.084 |
| Female sex, No. (%) | 19 (40.4%) | 28 (41.8%) | 1.000 |
| **Prior comorbidities, No. (%)** |  |  |  |
| Premorbid mRS, median (IQR) | 0 (0-2) | 0 (0-1) | 0.532 |
| Diabetes Mellitus | 19 (40.4.1%) | 19 (28.4%) | 0.227 |
| Antiplatelet medication | 13 (27.7%) | 19 (28.4%) | 1.000 |
| Oral anticoagulation | 11 (23.4%) | 19 (28.4%) | 0.667 |
| **Admission status, median (IQR)** |  |  |  |
| Glasgow Coma Scale | 8 (3-12) | 11 (3-14) | 0.241 |
| NIHSS | 22 (13-38) | 19 (12-38) | 0.679 |
| ICH Score | 2 (2-3) | 2 (1-3) | 0.377 |
| CHADS VASc Score | 3 (2-5) | 3 (2-5) | 0.894 |
| HAS Bled Score | 2 (2-4) | 3 (2-4) | 0.691 |
| **Imaging, median (IQR)** |  |  |  |
| Initial ICH volume, mL | 15.7 (6.6-35.45) | 18.6 (4.53-39.8) | 0.986 |
| ICH location, No. (%) |  |  |  |
| Deep | 28 (59.6%) | 37 (55.2%) | 0.703 |
| Lobar | 10 (21.3%) | 20 (29.9%) | 0.389 |
| Cerebellar | 7 (14.9%) | 7 (10.4%) | 0.566 |
| Brainstem | 2 (4.3%) | 3 (4.5%) | 1.000 |
| Intraventricular hemorrhage, No. (%) | 36 (76.6%) | 42 (62.7%) | 0.152 |
| Graeb Score | 4 (1-8) | 3 (0-8) | 0.309 |
| **Clinical parameters, No. (%)** |  |  |  |
| Mechanical ventilation | 43 (91.5%) | 60 (89.6%) | 1.000 |
| EVD † | 37 (78.7%) | 40 (59.7%) | 0.042 |

**Abbreviations:** EEN, early enteral nutrition; EVD, external ventricular drain; HAS-BLED, Hypertension, Abnormal Renal and Liver Function, Stroke, Bleeding, Labile INR-Measures, Elderly (age >65 y) and Drugs or Alcohol; ICH, intracerebral hemorrhage; IQR, interquartile range; mRS, modified Rankin Scale; nEEN, no early enteral nutrition; NIHSS, National Institutes of Health Stroke Scale; PS, propensity score.
